# Incidence of Myelodysplastic Syndrome in Japan

**DOI:** 10.2188/jea.JE20140042

**Published:** 2014-11-05

**Authors:** Dai Chihara, Hidemi Ito, Kota Katanoda, Akiko Shibata, Tomohiro Matsuda, Tomotaka Sobue, Keitaro Matsuo

**Affiliations:** 1 Division of Epidemiology and Prevention, Aichi Cancer Center Research Institute, Nagoya, Japan; 1 愛知県がんセンター研究所 疫学・予防部; 2 Surveillance Division, Center for Cancer Control and Information Services, National Cancer Center, Japan; 2 国立がん研究センター がん対策情報センター がん統計研究部; 3 Department of Environmental Medicine and Population Science, Osaka University Graduate School of Medicine, Osaka, Japan; 3 大阪大学大学院 医学系研究科 社会環境医学; 4 Department of Preventive Medicine, Kyushu University Faculty of Medical Science, Fukuoka, Japan; 4 九州大学大学院 医学系学府 予防医学分野

**Keywords:** myelodysplastic syndrome, MDS, incidence, epidemiology, Asia

## Abstract

**Background:**

Myelodysplastic syndromes (MDS) are a diverse group of clonal hematopoietic stem cell malignancies that primarily affect the elderly. Although the incidence of MDS in western countries has been well investigated, little is known about the incidence in Asian populations.

**Methods:**

To identify the incidence of MDS in Japan, we used population-based registry data from 1993 to 2008. The data represented 33.1% of the Japanese population. A total of 7995 patients were reported to the registry with newly diagnosed MDS during the study period.

**Results:**

Median age at diagnosis was 76 years. Incidence sharply increased with age, particularly in those aged over 70 years. The most recent crude incidence rate of MDS was 3.8 (95% CI 3.6–4.1) cases per 100 000 for men and 2.4 (95% CI 2.2–2.6) cases per 100 000 for women in 2008. Age-adjusted incidences of MDS in 2008, standardized by the world standard population, were 1.6 and 0.8 cases per 100 000 for men and women, respectively, while incidences standardized by the 1985 Japanese population were 2.5 and 1.2 cases per 100 000 for men and women, respectively.

**Conclusions:**

Our study reveals that some elderly patients suffer from MDS in Japan, but the incidence is lower than in Western countries. In current clinical practice, many elderly MDS patients are treated with supportive therapy based on an incomplete diagnosis, suggesting that the incidence estimated in this study might still be substantially underestimated. Accurate evaluation of the health impact of MDS requires evaluation of the mortality of the disease, continued surveillance, and improvement in the quality of cancer registry data.

## Abstract (ja)

**【背景】:**

骨髄異形成症候群(Myelodysplastic syndrome: MDS)は多能性幹細胞の様々な異常を元に、主に高齢者に発症する疾患である。欧米におけるMDSの罹患率に関する報告は散見されるが、アジア人における報告は乏しい。

**【方法】:**

日本におけるMDSの罹患率を算出するため、1993年から2008年までの地域がん登録データを使用した。データは日本人口の33.1%をカバーするものであり、合計で7995人のMDSの患者が研究期間中データに登録された。

**【結果】:**

登録されたMDSの年齢中央値は76歳であった。罹患率は年齢とともに上昇し、特に70歳以上で急激に上昇していた。最も直近のデータである2008年の粗罹患率は男性で10万人年あたり3.8人（95%信頼区間：3.6-4.1人）、女性で10万人年あたり2.4人（95%信頼区間：2.2-2.6人）であった。世界標準人口を用いた2008年の年齢調整罹患率は男性で10万人年あたり1.6人、女性で10万人年あたり0.8人であり、1985年の日本人口を用いた2008年の年齢調整罹患率は10万人年あたり2.5人、女性で10万人年あたり1.2人であった。

**【結語】:**

日本人において、高齢者でMDS罹患率が高い事が明らかになった。また、本邦におけるMDS罹患率は欧米諸国の報告のものよりも低かった。現在の臨床現場における高齢者MDSに対する診断・治療状況を鑑みるに、今回算出した罹患率は実際よりも過小評価されている可能性が考えられる。MDSによる健康影響のより正確な把握には、死亡率の検討を含むサーベイランス、地域がん登録の精度向上が必要と考えられる。

## INTRODUCTION

Myelodysplastic syndromes (MDS) are a diverse group of clonal hematopoietic stem cell malignancies in the elderly that present with persistent bone marrow failure and peripheral blood cytopenias. Approximately 30% of patients progress to acute myeloid leukemia, which is always fatal in the elderly.^1^^,^^2^ Population-based registry data in the United States (U.S.) and Europe indicate an incidence of MDS in western countries of around 2 to 4 cases per 100 000 person-years with male predominance.^3^^,^^4^ The descriptive epidemiology of MDS has been well investigated in western countries^3^^–^^8^ but not in Asian populations, partly because of the scarcity of population-based registry data for MDS in Asia. A U.S. study showed that incidence differed by race,^3^ indicating that reference data in Asians based on population-based data would be of interest.

Several studies have reported the characteristics of MDS in Asian populations.^9^^–^^12^ Matsuda et al reported differences in the characteristics of MDS between patients in Japan and Germany, in which they found that the patients’ median age of 57 years in Japan was significantly younger than the median age of 71 years in German patients.^11^ Median age at diagnosis of other reports from Asia was 60 and 62 years, which also suggest younger age of MDS in an Asian population.^9^^,^^12^ However, these studies were conducted using a case-series design, and their data were not population-based. Patients in case series rarely represent general patient populations, and their data cannot be used to estimate incidence in a population.

Here, we evaluated the incidence and trends in MDS in Japan using population-based registry data in Japan.

## METHODS

We used population-based cancer registry data from the Monitoring of Cancer Incidence in Japan (MCIJ) project, which was started in 2007 as a national project that aimed to collect and unite the cancer registry data of each of Japan’s prefectures using a standardized protocol. MCIJ data have previously been used to estimate annual cancer incidences in Japan.^13^^,^^14^ For the quality control, prefectures that reported death certificate notices (DCN) of less than 50% were included in the study. Thus, we used the data of 16 prefectures (Iwate, Yamagata, Niigata, Tochigi, Chiba, Kanagawa, Toyama, Fukui, Aichi, Shiga, Tottori, Okayama, Hiroshima, Saga, Nagasaki, and Kumamoto), which are included in the 2008 MCIJ data. These data represent 33.1% of the Japanese population.^14^

The period covered in this analysis was 1993 to 2008. In the Japanese cancer registry systems, disease incidence data are constructed and collected according to the International Classification of Diseases for Oncology (ICD-O) coding. The ICD-O-2 code was used up to and including 2002, while the ICD-O-3 code has been used since 2003. In the ICD-O-2, MDS was not considered a malignant disease but was rather coded as a neoplasm with uncertainty over whether it is in fact malignant, such that MDS was incidentally reported based on the definition of the local cancer registry. For the ICD-O-3, however, MDS was reclassified as a malignant disease and coded as 9980–9989. MDS coded by ICD-O-2 before 2002 have been re-coded in ICD-O-3 in each registry, so for the present study, we extracted MDS cases from the data using the ICD-O-3 code.

Incidence rates were standardized by age-adjustment to the world standard population and also to the 1985 Japanese model population, and rates were calculated for the number of newly diagnosed cases of MDS per 100 000 person-years as described in detail elsewhere.^15^ All analyses were performed with STATA version 11 (STATA Corporation, College Station, TX, USA).

## RESULTS

**Figure 1.**
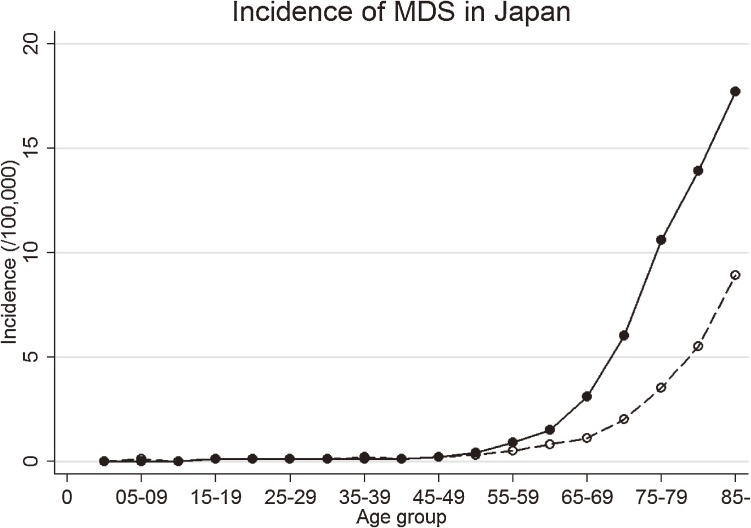
Age-specific incidence of MDS in Japan. Circles indicate the observed incidence, connected by lines. Solid lines and solid circles are for men, and dashed lines and hollow circles for women.

A total of 7995 patients were reported to the data registries of 16 prefectures with newly diagnosed MDS from 1993 to 2008. The proportion of DCN cases were 46.6%. Median age at diagnosis was 76 years (interquartile range 68–82 years). Incidence sharply increased with age, particularly in those over age 70 years (Figure 1), with 3.1 cases per 100 000 men and 1.1 cases per 100 000 women in those aged 65–69 years versus 17.7 and 8.9 cases per 100 000, respectively, in those aged over 85 years.

**Figure 2.**
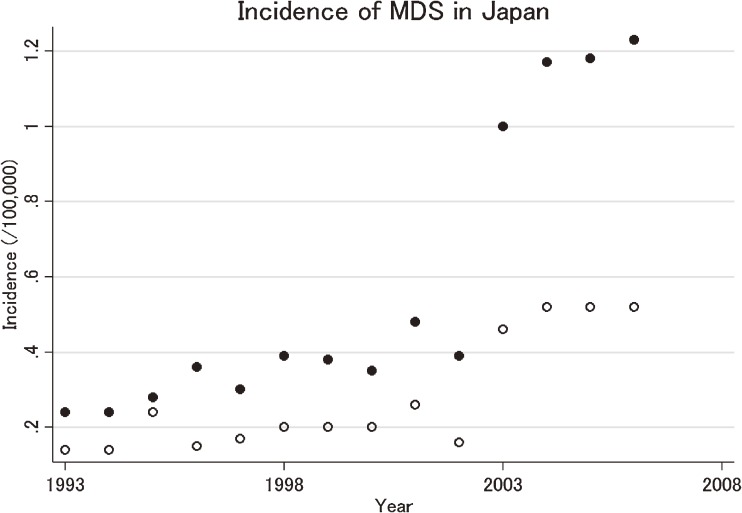
Incidence and trends of MDS in Japan. Circles indicate the observed age-standardized incidence. Solid circles are for men, and hollow circles are for women.

Estimated annual age-standardized incidences of MDS during the study period are shown as circles in Figure 2, and the exact rates with 95% confidence intervals (CIs), which are the basis of Figure 2, are summarized in Table. Incidence showed male predominance, with nearly twice as many cases in men as in women. As shown in Figure 2, incidence significantly differed between 2002 and 2003, the year that the cancer registries in Japan started to use ICD-O-3. The most recent crude incidence rate of MDS is 3.8 (95% CI 3.6–4.1) per 100 000 for men and 2.4 (95% CI 2.2–2.6) per 100 000 for women in 2008. Age-adjusted incidences of MDS in 2008, standardized by the world standard population, were 1.6 and 0.8 cases per 100 000 for men and women, respectively, while incidences standardized by the 1985 Japanese population were 2.5 and 1.2 cases per 100 000 for men and women, respectively.

**Table.** Age-standardized incidence of MDS

Year	1993	1994	1995	1996	1997	1998	1999	2000	2001	2002	2003	2004	2005	2006	2007	2008
Men																
Age-standardized rate-W (95% CI)	0.22 (0.21–0.22)	0.22 (0.21–0.23)	0.31 (0.30–0.32)	0.29 (0.28–0.30)	0.27 (0.26–0.28)	0.28 (0.27–0.29)	0.31 (0.30–0.32)	0.26 (0.26–0.27)	0.39 (0.39–0.40)	0.35 (0.34–0.35)	0.95 (0.94–1.00)	1.12 (1.11–1.13)	1.20 (1.19–1.22)	1.33 (1.32–1.34)	1.71 (1.70–1.72)	1.61 (1.60–1.62)
Age-standardized rate-J (95% CI)	0.30 (0.29–0.31)	0.33 (0.32–0.34)	0.43 (0.42–0.44)	0.38 (0.38–0.39)	0.40 (0.40–0.41)	0.41 (0.40–0.42)	0.44 (0.43–0.45)	0.37 (0.36–0.38)	0.58 (0.57–0.59)	0.51 (0.50–0.52)	1.43 (1.41–1.44)	1.72 (1.71–1.73)	1.81 (1.79–1.82)	2.03 (2.02–2.05)	2.54 (2.52–2.56)	2.46 (2.44–2.47)
Women																
Age-standardized rate-W (95% CI)	0.15 (0.14–0.15)	0.14 (0.13–0.14)	0.24 (0.23–0.25)	0.12 (0.12–0.13)	0.13 (0.12–0.13)	0.17 (0.17–0.18)	0.17 (0.17–0.18)	0.19 (0.18–0.19)	0.22 (0.22–0.23)	0.16 (0.16–0.16)	0.46 (0.45–0.46)	0.55 (0.54–0.56)	0.54 (0.53–0.54)	0.55 (0.54–0.55)	0.75 (0.74–0.76)	0.82 (0.82–0.83)
Age-standardized rate-J (95% CI)	0.19 (0.18–0.20)	0.18 (0.17–0.18)	0.31 (0.30–0.31)	0.17 (0.16–0.18)	0.19 (0.18–0.20)	0.23 (0.22–0.24)	0.23 (0.23–0.24)	0.26 (0.25–0.26)	0.31 (0.30–0.32)	0.24 (0.23–0.25)	0.66 (0.65–0.67)	0.80 (0.79–0.81)	0.78 (0.77–0.79)	0.82 (0.81–0.83)	1.07 (1.06–1.08)	1.19 (1.18–1.20)

Abbreviations: CI, confidence interval; MDS, myelodysplastic syndrome.

Incidences are standardized by world populations-W (/100 000 people) and Japanese 1985 population-J (/100 000 people).

We initially aimed to evaluate MDS subtypes in detail, but 73% of cases in the registry were coded as MDS-not otherwise specified (code: 9989), leaving too few cases to evaluate incidence and trends by subtype.

## DISCUSSION

The most recently reported age-adjusted incidence of MDS using the world population, of 1.6 cases per 100 000 for men and 0.8 cases for women in 2008, is less than half that in the U.S. and close to that in China.^3^^,^^12^ This difference among countries and races might suggest differences in risk exposure among countries or in genetic susceptibility among races. One of the most reliable cancer registries is the Surveillance Epidemiology and End Results (SEER), a population-based registry in the U.S., but a recent U.S. study reported that use of the SEER data resulted in a marked underestimation of the true incidence of MDS.^16^ Many patients with MDS are diagnosed at an advanced age and accordingly are no longer candidates for aggressive treatment such as allogeneic bone marrow transplant, which is the only treatment with curative potential. Instead, older MDS patients are offered supportive therapy with transfusion to maintain their quality of life. Except for the DNA-hypomethylating agents,^17^ cytotoxic chemotherapy does not prolong survival, so even the thorough evaluation and accurate diagnosis of MDS may not lead to effective treatment that results in a meaningful improvement in prognosis. Thus, it is possible that many elderly patients with cytopenia have not received sufficient diagnostic evaluation and have therefore not been diagnosed with MDS. In the U.S. study, the estimated incidence of MDS in elderly patients, using a new algorithm based on claims in the Medicare system, was nearly four-fold that estimated using the SEER data.^16^ Similar underestimation of incidence might also occur with the Japanese registry data. To get a more accurate understanding of the public health impact of MDS in Japan and reflect findings in public health policy and future research, more attention must be paid to the cancer registry system in Japan.

Previous studies of MDS based on case-series from Japan reported a median age at diagnosis of 57–60 years, which was significantly younger than in western countries.^9^^,^^11^ In contrast, the median age in the present study was 76 years, which is similar to that in western countries. Since cases in previous Japanese studies were collected at a centralized hospital, their diagnosis may reflect a degree of bias toward younger patients who required aggressive treatment.

We observed a dramatic increase in the incidence of MDS from 2002 to 2003, but this indicates a certain incompleteness in the early registry data of MDS (Figure 2) and is highly likely to be due to a change in the coding rule at this time. After the coding rule changed, incidence seems to have gradually increased in Japan since 2004. However, the reason for the increase in the incidence of MDS in Japan since 2004 requires further investigation, although one reason could be an artificial increase subjected to the change in the coding rule in 2003. Gradual and consistent acclimation to the new coding rule may have lead to increased numbers of reported MDS cases. In addition, the Japanese government started to prepare designated cancer care hospitals to provide better cancer care in the early 2000s, and many of the candidate hospitals antecedently launched cancer registration within their institutions.^18^^,^^19^ This might have contributed to an increase in the number and the quality of reported MDS cases. Several factors are known to increase the risk of MDS, including previous exposure to chemotherapy and radiotherapy, as well as obesity and smoking.^20^^–^^22^ Although exposure to these risks in the Japanese population appears to be increased or increasing,^14^^,^^23^ the increasing trend of MDS revealed in this study should be interpreted with caution. Continued surveillance of registry data will enable us to evaluate the trend in the future, helping to quantify the degree of impact of the change in the coding rule on the increases in incidence.

We found male predominance in the incidence of MDS, which is consistent with previous studies.^3^^,^^10^ Smoking and obesity increase the risk of MDS, which may be one reason for the male predominance.^21^ The male-to-female ratio of cases of MDS is around 2 in Japan, which is slightly higher than that in Western countries.^3^^,^^4^ The reasons for a higher male-to-female ratio of cases of MDS in Japan are hard to elucidate, but a lower consultation rate in the elderly female population may result in a lower rate of accurate diagnosis of MDS in this group.

In conclusion, the present study shows that the median age at diagnosis of MDS in Japan was similar to or even higher than that in western countries, but the incidence rate was lower. Nevertheless, MDS is a disease of the elderly that seldom receives a thorough evaluation for diagnosis that substantial number of cases might have been dropped from the registry data, resulting in an underestimation of the real incidence of MDS in Japan. Accurate evaluation of the health impact of MDS in Japan requires evaluation of the mortality of the disease, continued surveillance, and improvement in the quality of the cancer registry data.

## ONLINE ONLY MATERIAL

Abstract in Japanese.

## References

[1] Swerdlow S, Campo E, Harris N, Jaffe E, Pileri S, Stein H, et al. WHO classification of Tumours of Haematopoietic and Lymphoid Tissues. Lyon, France: International Agency for Research on Cancer (IARC); 2008.

[2] Disperati P, Ichim CV, Tkachuk D, Chun K, Schuh AC, Wells RA. Progression of myelodysplasia to acute lymphoblastic leukaemia: implications for disease biology. Leuk Res. 2006;30(2):233–9. 10.1016/j.leukres.2005.06.011 16046234

[3] Rollison DE, Howlader N, Smith MT, Strom SS, Merritt WD, Ries LA, . Epidemiology of myelodysplastic syndromes and chronic myeloproliferative disorders in the United States, 2001–2004, using data from the NAACCR and SEER programs. Blood. 2008;112(1):45–52. 10.1182/blood-2008-01-134858 18443215

[4] Sant M, Allemani C, Tereanu C, De Angelis R, Capocaccia R, Visser O, . Incidence of hematologic malignancies in Europe by morphologic subtype: results of the HAEMACARE project. Blood. 2010;116(19):3724–34. 10.1182/blood-2010-05-282632 20664057

[5] Germing U, Strupp C, Kündgen A, Bowen D, Aul C, Haas R, . No increase in age-specific incidence of myelodysplastic syndromes. Haematologica. 2004;89(8):905–10. 15339672

[6] Ma X, Does M, Raza A, Mayne ST. Myelodysplastic syndromes: incidence and survival in the United States. Cancer. 2007;109(8):1536–42. 10.1002/cncr.22570 17345612

[7] Goldberg SL, Chen E, Corral M, Guo A, Mody-Patel N, Pecora AL, . Incidence and clinical complications of myelodysplastic syndromes among United States Medicare beneficiaries. J Clin Oncol. 2010;28(17):2847–52. 10.1200/JCO.2009.25.2395 20421543

[8] Neukirchen J, Schoonen WM, Strupp C, Gattermann N, Aul C, Haas R, . Incidence and prevalence of myelodysplastic syndromes: data from the Dusseldorf MDS-registry. Leuk Res. 2011;35(12):1591–6. 10.1016/j.leukres.2011.06.001 21708407

[9] Oguma S, Yoshida Y, Uchino H, Maekawa T, Nomura T, Mizoguchi H. Clinical characteristics of Japanese patients with primary myelodysplastic syndromes: a co-operative study based on 838 cases. Anemia Study Group of the Ministry of Health and Welfare. Leuk Res. 1995;19(3):219–25. 10.1016/0145-2126(94)00135-W 7700083

[10] Shimizu H, Matsushita Y, Aoki K, Nomura T, Yoshida Y, Mizoguchi H. Prevalence of the myelodysplastic syndromes in Japan. Int J Hematol. 1995;61(1):17–22. 10.1016/0925-5710(94)00339-G 7718765

[11] Matsuda A, Germing U, Jinnai I, Misumi M, Kuendgen A, Knipp S, . Difference in clinical features between Japanese and German patients with refractory anemia in myelodysplastic syndromes. Blood. 2005;106(8):2633–40. 10.1182/blood-2005-01-0040 15972453

[12] Wang W, Wang H, Wang XQ, Lin GW. First report of incidence of adult myelodysplastic syndrome in China. Ann Hematol. 2012;91(8):1321–2. 10.1007/s00277-011-1389-7 22194142

[13] Matsuda T, Marugame T, Kamo K, Katanoda K, Ajiki W, Sobue T; Japan Cancer Surveillance Research Group. Cancer incidence and incidence rates in Japan in 2006: based on data from 15 population-based cancer registries in the monitoring of cancer incidence in Japan (MCIJ) project. Jpn J Clin Oncol. 2012;42(2):139–47. 10.1093/jjco/hyr184 22172347

[14] Matsuda A, Matsuda T, Shibata A, Katanoda K, Sobue T, Nishimoto H; Japan Cancer Surveillance Research Group. Cancer incidence and incidence rates in Japan in 2007: a study of 21 population-based cancer registries for the Monitoring of Cancer Incidence in Japan (MCIJ) project. Jpn J Clin Oncol. 2013;43(3):328–36. 10.1093/jjco/hys233 23296772

[15] Chihara D, Ito H, Matsuda T, Shibata A, Katsumi A, Nakamura S, . Differences in incidence and trends of haematological malignancies in Japan and the United States. Br J Haematol. 2014;164(4):536–45. 10.1111/bjh.12659 24245986 PMC3907701

[16] Cogle CR, Craig BM, Rollison DE, List AF. Incidence of the myelodysplastic syndromes using a novel claims-based algorithm: high number of uncaptured cases by cancer registries. Blood. 2011;117(26):7121–5. 10.1182/blood-2011-02-337964 21531980 PMC3143554

[17] Fenaux P, Mufti GJ, Hellstrom-Lindberg E, Santini V, Finelli C, Giagounidis A, . Efficacy of azacitidine compared with that of conventional care regimens in the treatment of higher-risk myelodysplastic syndromes: a randomised, open-label, phase III study. Lancet Oncol. 2009;10(3):223–32. 10.1016/S1470-2045(09)70003-8 19230772 PMC4086808

[18] Higashi T, Nakamura F, Shibata A, Emori Y, Nishimoto H. The National Database of Hospital-based Cancer Registries: A Nationwide Infrastructure to Support Evidence-based Cancer Care and Cancer Control Policy in Japan. Jpn J Clin Oncol. 2014;44(1):2–8. 10.1093/jjco/hyt013 23448800

[19] Katanoda K, Ajiki W, Matsuda T, Nishino Y, Shibata A, Fujita M, . Trend analysis of cancer incidence in Japan using data from selected population-based cancer registries. Cancer Sci. 2012;103(2):360–8. 10.1111/j.1349-7006.2011.02145.x 22066698

[20] Du Y, Fryzek J, Sekeres MA, Taioli E. Smoking and alcohol intake as risk factors for myelodysplastic syndromes (MDS). Leuk Res. 2010;34(1):1–5. 10.1016/j.leukres.2009.08.006 19747728

[21] Ma X, Lim U, Park Y, Mayne ST, Wang R, Hartge P, . Obesity, lifestyle factors, and risk of myelodysplastic syndromes in a large US cohort. Am J Epidemiol. 2009;169(12):1492–9. 10.1093/aje/kwp074 19395696 PMC2727203

[22] Kantarjian HM, Keating MJ. Therapy-related leukemia and myelodysplastic syndrome. Semin Oncol. 1987;14(4):435–43. 3321448

[23] Ministry of Health, Labour and Wealfare. http://www.mhlw.go.jp/

