# Supplementary material for: Incidence of Myelodysplastic Syndrome in Japan
Source: J Epidemiol. 2014 Nov 5;24(6):469–73. doi: 10.2188/jea.JE20140042 (PMC4213221; doi:10.2188/jea.JE20140042)
Supplement: Abstract in Japanese. [file je-24-469-s001.pdf]

日本における骨髄異形成症候群の罹患率

千原　大[1]、伊藤　秀美[1]、片野田　耕太[2]、柴田　亜希子[2]、松田　智大[2]、祖父江　友孝[3]、松尾　恵太郎[4]

1. 愛知県がんセンター研究所　疫学・予防部
2. 国立がん研究センター　がん対策情報センター　がん統計研究部
3. 大阪大学大学院　医学系研究科　社会環境医学
4. 九州大学大学院　医学系学府　予防医学分野

【背景】骨髄異形成症候群(Myelodysplastic syndrome: MDS)は多能性幹細胞の様々な異常を元に、主に高齢者に発症する疾患である。欧米における MDS の罹患率に関する報告は散見されるが、アジア人における報告は乏しい。
【方法】日本における MDS の罹患率を算出するため、1993 年から 2008 年までの地域がん登録データを使用した。データは日本人口の 33.1%をカバーするものであり、合計で 7995 人の MDS の患者が研究期間中データに登録された。
【結果】登録された MDS の年齢中央値は 76 歳であった。罹患率は年齢とともに上昇し、特に 70 歳以上で急激に上昇していた。最も直近のデータである 2008 年の粗罹患率は男性で 10 万人年あたり 3.8 人（95%信頼区間 : 3.6-4.1 人）、女性で 10 万人年あたり 2.4 人（95%信頼区間 : 2.2-2.6 人）であった。世界標準人口を用いた 2008 年の年齢調整罹患率は男性で 10 万人年あたり 1.6 人、女性で 10 万人年あたり 0.8 人であり、1985 年の日本人口を用いた 2008 年の年齢調整罹患率は 10 万人年あたり 2.5 人、女性で 10 万人年あたり 1.2 人であった。
【結語】日本人において、高齢者で MDS 罹患率が高い事が明らかになった。また、本邦における MDS 罹患率は欧米諸国の報告のものよりも低かった。現在の臨床現場における高齢者 MDS に対する診断・治療状況を鑑みるに、今回算出した罹患率は実際よりも過小評価されている可能性が考えられる。MDS による健康影響のより正確な把握には、死亡率の検討を含むサーベイランス、地域がん登録の精度向上が必要と考えられる。

キーワード : 骨髄異形成症候群、MDS、罹患率、疫学、アジア
